# Factors Influencing the Development and Severity of Cognitive Decline in Patients with Chronic Heart Failure

**DOI:** 10.3390/medicina60111859

**Published:** 2024-11-13

**Authors:** Marius Militaru, Daniel Florin Lighezan, Cristina Tudoran, Mariana Tudoran, Anda Gabriela Militaru

**Affiliations:** 1 Department VIII, Neuroscience, Discipline of Neurology II, University of Medicine and Pharmacy “Victor Babes” Timisoara, E. Murgu Square, Nr. 2, 300041 Timisoara, Romania; marius.militaru@umft.ro; 2 Municipal Emergency Hospital Timisoara, Gheorghe Dima Street Nr. 5, 300254 Timisoara, Romania; dlighezan@umft.ro (D.F.L.); militaru.anda@umft.ro (A.G.M.); 3 Center of Advanced Research in Cardiology and Hemostasology, University of Medicine and Pharmacy “Victor Babes” Timisoara, E. Murgu Square, Nr. 2, 300041 Timisoara, Romania; 4 Department V, Internal Medicine I, Discipline of Medical Semiology I, University of Medicine and Pharmacy “Victor Babes” Timisoara, E. Murgu Square, Nr. 2, 300041 Timisoara, Romania; 5 Department VII, Internal Medicine II, Discipline of Cardiology, University of Medicine and Pharmacy “Victor Babes” Timisoara, E. Murgu Square, Nr. 2, 300041 Timisoara, Romania; 6 Center of Molecular Research in Nephrology and Vascular Disease, University of Medicine and Pharmacy “Victor Babes” Timisoara, E. Murgu Square, Nr. 2, 300041 Timisoara, Romania; 7 County Emergency Hospital “Pius Brinzeu”, L. Rebreanu Street, Nr. 156, 300723 Timisoara, Romania; mariana.tudoran@gmail.com

**Keywords:** cognitive decline, chronic heart failure, dementia, neuropsychological evaluation scales, left-ventricular ejection fraction

## Abstract

*Background and Objectives*: Considering the increasing prevalence of chronic heart failure (CHF) and cognitive decline (CD) observed in recent decades and the complex interrelation between these two pathologies often encountered in the same patient, in this study, we aimed to highlight the connection between CHF, defined as recommended by the European Society of Cardiology guidelines, and CD, evaluated by employing five neuropsychological scales. *Materials and Methods*: Our study was conducted on 190 patients with very high cardiovascular risk profiles admitted between 5 September 2021 and 15 November 2023 in the Municipal Emergency Hospital Timisoara. Of these, 103 had CHF (group A) and 87 did not (group B). *Results*: Although similar concerning age, sex distribution, and risk factors (excepting lipid profile), patients from group A had lower Mini Mental State Evaluation (MMSE) and Montreal Cognitive Assessment (MoCA levels (*p* = 0.003, respectively, *p* = 0.017) scores, more reduced daily activity (*p* = 0.021), and more severe depression (*p* = 0.015) compared to group B. We documented statistically significant correlations between left-ventricular ejection fraction (LVEF) and the levels of N-terminal pro–B-type natriuretic peptide (NT-pro-BNP), as well as with the results of MMSE (r = 0.226, *p* = 0.002 and r = −0.275, *p* = 0.005, respectively), daily activity, and depression (*p* ˂ 0.001). Multi-logistic regression models indicated age, blood pressure values, decreased daily activity, and depression as risk factors for CD in patients with CHF. *Conclusions*: In patients with CHF, there is an increased propensity of CD, with a direct relationship between MMSE and LVEF levels and an indirect one between MMSE and NT-pro-BNP levels. The concomitance of depression and reduced activity levels are aggravating CD in these patients.

## 1. Introduction

In recent decades, chronic heart failure (CHF) has evolved into a global healthcare problem, probably due to longer life expectancies and the development of novel pharmacological agents for the treatment of cardiovascular (CV) pathologies, these also aiming to alleviate symptoms and improve cardiac performance in CHF. These new CHF therapies have significantly reduced hospitalization duration and frequency, and have increased patients’ survival. In the context of CHF, the improvement in left-ventricular ejection fraction (LVEF) after targeted pharmacological treatment has led to a new definition of CHF, known as heart failure with improved ejection fraction (HFimpEF) [1]. For these patients, a key concern is their ability to adhere to the therapy, adopt lifestyle changes, and take care of themselves [2,3]. Recently, telemedicine has been a significant aid in this regard, also helping to reduce healthcare costs associated with patient care [4].

Although CHF develops as a consequence of underlying CV diseases, often associated with other pathologies and risk factors, an increased prevalence of cognitive decline (CD) of around 40% has been observed in these patients, reaching even 60% in older individuals [2]. It has also been documented that CD develops earlier in patients with CHF, contributing to elevated morbidity and mortality [2].

The underlying mechanisms responsible for the association between CHF and CD are still largely unknown. Some studies suggest that diminished cerebral blood flow, inflammatory processes, endothelial dysfunction, atherosclerosis, protein abnormalities, and thromboembolic events, especially in patients with atrial fibrillation (AF), could be causes of CD [5,6]. In patients with CHF and CD, this relationship is bidirectional; impaired cognition reduces their compliance to diet and lifestyle-changing measures and their ability to manage their medication [5,7]. It has been observed that among patients with low treatment compliance, more than half had cognitive disorders [8]. Even more so, an altered mental status in patients with CHF reduces their independence and ability to take care of themselves and increases the need for caregivers, leading to higher costs for the healthcare system.

This is why the diagnosis of early signs of CD or even dementia became very important in patients with CHF [9]. Several studies have employed neuropsychological evaluation scales, such as the Montreal Cognitive Assessment (MoCA) or the Mini Mental State Examination (MMSE) scale, to diagnose CD, and to follow up its evolution [10]. While most research has concentrated on the severity of the cognitive impairment, less emphasis has been given to the contribution of other contributing factors, such as depression, the limitation of daily activity, and self-care ability, on the development and progression of CD and CHF [4,5]. Various scales can quantify these disturbances and allow their follow-up, with this most important when treating patients with CHF and cognitive impairment [5,6].

On the other hand, serious effort has been invested in determining which parameters defining CHF could be associated with a higher risk of developing CD [11,12]. Another study demonstrated a relationship between age and the severity of CHF in patients with this pathology and CD [10]. In a more recent study, Giacona et al. documented that decreased LVEF, increased left-ventricular hypertrophy (LVH), and concentric remodeling have a negative impact on cognitive function [13].

According to the European Guidelines on Heart Failure [14,15], N-terminal pro–B-type natriuretic peptide (NT-pro-BNP) is mandatory to establish the diagnosis of CHF with preserved LVEF. Several studies have assessed the relationship between NT-pro-BNP and early signs of cognitive impairment or dementia [16,17]. It has been suggested that the severity of CHF, expressed through an increased NT-pro-BNP, was associated with an augmented risk of dementia in elderly individuals [16]. Another study demonstrated an association between NT-pro-BNP levels and impairments of cerebral small vessels on brain magnetic resonance imaging (MRI) [18]. A recent study indicated that even an increase in NT-pro-BNP levels was independently associated with the onset of dementia and correlated better with CD than baseline NT-pro-BNP levels [19]. These studies indicate that NT-pro-BNP levels could be used to detect patients with CV pathology who presented a higher risk of developing dementia, and who would benefit from an earlier diagnosis and adequate treatment of CD [17]. Because of the increased healthcare burden represented by CD in CHF patients and the fact that both conditions cannot be cured, only treated, their prevention is of the utmost importance.

Considering that the majority of the existing studies analyzed the interdependence of CHF and CD, only concerning the cognitive domain, in this study we intended to highlight the bidirectional impact of depression and a limited self-care capacity, often encountered in patients with CHF, in relation to the occurrence and severity of CD. A second aim was to assess the contribution of several factors (such as age, the severity of some parameters characterizing cardiac function, and comorbid depression) on the higher odds and severity of CD, diagnosed according to the results of multiple neuropsychological tests in patients with CHF.

## 2. Materials and Methods

### 2.1. Study Population and Clinical Procedures

**Figure 1:**
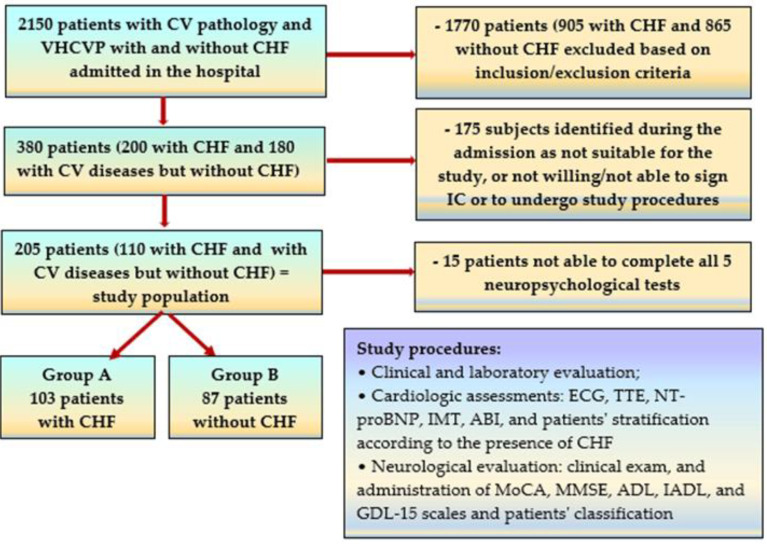
The flow chart for the selection process of the patients’ group.

For our study, from 2150 patients with an established atherosclerotic CV disease and/or a high CV risk profile (CVRP), defined by a score over 15%, as stated by the European Society of Cardiology [20], admitted between 5 September 2021 and 15 November 2023 in the Neurology and Internal Medicine departments of the Municipal Emergency Hospital Timisoara for neurologic or CV pathology, and after excluding 1960 individuals for various reasons (see inclusion and exclusion criteria), we selected a total number of 190 patients, who lacked an anterior diagnosis of CD or dementia, as illustrated in Figure 1.

According to the presence or absence of CHF, we divided our patients’ group into 2 subcategories: group A—103 subjects diagnosed with CHF, and group B—87 age-matched patients with a very high CV risk, but without a diagnosis of CHF. The diagnosis and classification of CHF were performed according to the European Guidelines recommendations based on the presence of signs and symptoms suggestive of CHF, the level of NT-pro-BNP (over 300 pg/mL in patients with sinus rhythm and over 600 pg/mL in those with AF), the presence of structural cardiac alterations, and reduced, mid-range, or preserved LVEF on transthoracic echocardiography (TTE) [14,15]. All patients from group A were already known to have had CHF for 6 to 60 months before our study and were on optimal medical therapy (angiotensin-converting enzyme inhibitors/angiotensin receptor blockers/sacubitril/valsartan, beta-blockers, mineralocorticoid receptor antagonists, sodium-glucose cotransporter 2 inhibitors, and diuretics), in accordance with the guidelines’ recommendation [14,15]. In total, 58 patients from group A had AF and were treated with new oral anticoagulants (22 with Apixaban, 16 with Rivaroxaban, 12 with Dabigatran, and 8 with Edoxaban). None of our subjects was treated with Acenocumarol or Warfarin to reduce the bleeding risk. Of the remaining 55 subjects from group A, 37 were treated with Aspirin, and 18 with Clopidogrel. In group B, 56 patients received Aspirin, and 31 Clopidogrel.

Inclusion criteria: (1) aged over 45 years, considering the reduced probability of having a very high CV risk profile of over 15%, developing cognitive impairment, and suffering from CHF before this age; (2) subjects with signs of cognitive impairment, but without a previous neurological assessment of their cognitive status, diagnosis, or therapy for CD or dementia; (3) newly diagnosed cases of CD—those currently hospitalized in the Municipal Hospital, without having received treatment for this pathology; (4) a history of CV disease; (5) patients with elevated CVRP.

Exclusion criteria: (1) patients unable or not willing to give their informed consent; (2) decompensated CHF, and other acute CV pathologies, such as acute myocardial infarction and acute coronary syndrome.; (4) systolic blood pressure (SBP) levels over 180 mmHg, and/or diastolic blood pressure (DBP) over 130 mmHg; (5) patients who refused to comply with the indicated lifestyle measures and medication; (6) chronic alcohol and drug abuse; (7) history of significant mental health disturbances (schizophrenia, paranoia, post-traumatic stress disorder, severe forms of depression and anxiety requiring treatment); (8) subjects already diagnosed with CD and under therapy for this condition; (9) medical history of severe depression.

During hospitalization, all subjects from this study underwent a comprehensive clinical examination, followed by cardiological and neurological assessments, and N-terminal pro–B-type natriuretic peptide (NT-pro-BNP) levels were determined. During the clinical assessment, the medical history, SBP, DBP, and heart rate (HR) were recorded. Demographic and laboratory data, such as those regarding the lipid profile, and renal and liver function parameters, were obtained for all subjects from the hospital’s database, to diagnose other associated CV pathologies. An electrocardiogram (ECG) and a Doppler carotid artery ultrasound were performed on every patient to assess the presence of arrhythmias, atherosclerotic plaques, and to calculate intima–media thickness (IMT). The IMT was determined at the distal wall of both common carotid arteries, at 1 cm from the carotid bulb, with a General Electric Vivid E9 ultrasound system with a 9L MHz transducer. An ankle−brachial index (ABI) was also determined. ABI is a quick, reproducible, noninvasive method to determine the presence of peripheral artery disease (PAD). The patient’s SBP at the level of the left and right arm was measured in the morning, at the bedside, followed by the SBP being measured at the level of the ankle, and the ratio between the two values was calculated; a value between 1.0 and 1.4 was considered normal, while an ABI under 0.90 indicated PAD [21].

A TTE was performed in all patients following guideline recommendations with a General Electric Vivid E90 CISPR 11, Group 1, Class A ultrasound system (GE Vingmed Ultrasound AS, Strandpromenaden 45, 3191 Harten, Norway), with an M5S MHz transducer, in 2, 3, and 4-chamber view, to assess the left-ventricular (LV) systolic and diastolic performance, and calculate the LVEF [22]. All TTE and Doppler evaluations were performed by the same skilled examiner and on the same ultrasound machine.

### 2.2. Neuropsychological Tests

The existence and severity of CD were assessed by a senior specialist in neurology by employing several scales that are frequently used in healthcare units and available in Romania free of charge: MMSE, MoCA, Activities of Daily Living (ADL), Instrumental Activities of Daily Living (IADL), and the Geriatric Depression Scale (GDS-15). All these scales were applied by the first author of this study, who asked the questions, completed the answers, and, where needed, supervised the patient in accomplishing the task.

The MoCA instrument is widely used to assess cognitive decline. This method allows for the evaluation of multiple cognitive functions: attention, concentration, temporospatial orientation, visuospatial capacity, language, working memory, short- and long-term memory recall, executive functions, ability for fluent oral communication, and two-item oral abstraction tasks. This test′s completion takes approximately 10 min, a maximum score of 30 points can be achieved, and results below 26 points signify impairment [23].

The MMSE scale is widely used to identify alterations in cognitive function. MMSE is employed to quantify cognitive capacities such as attention, concentration, calculation, temporo-spatial orientation, and the ability to remember, construct, and manipulate words [21,22,23]. It assesses the evolution and severity of CD and analyses the progression of cognitive alterations over time. The results of this test should be appreciated according to patients’ age and level of education [24,25]. Thirty is the maximum score, and a score between 24 and 27 can indicate mild CD, and values under 24 indicate dementia [24,26].

The IADL scale is employed to evaluate the capacity of practicing current daily activities, which confer individual independence and permit patients to take care of themselves. It refers to 8 domains of activities necessary for a normal life: financial self-management, cleaning, cooking, shopping, etc. Each of them are scored from 0—meaning a low functionality—to a maximum of 8, this signifying normal functionality [27,28].

The ADL score is a common scale, often employed to evaluate the daily self-care of the patient [27]. The ADL scale takes about 10 min to apply and the scores range between 0 (indicating reduced functionality) and 10 (standing for high functionality), with 5 basic categories: dressing, eating, personal hygiene, transferring/mobility, and maintaining continence [24].

The GDS-15 is used to assess depression. As it is easier to administrate in patients with CD, in this study we employed the abbreviated variant with 15 questions (GDS-15) indicating the presence or absence of depression, with a sensitivity of 92% and a specificity of 89% [29,30]. GDS-15 is a short test, easy to apply, and takes approximately 10–15 min.

The MMSE and MoCA scales are commonly used to assess CD and dementia, offering complementary information for the diagnosis and evaluation of cognitive disorders. To appreciate the patient’s self-care ability, we employed the ADL and IADL scales, which are available online, in Romanian language, free of charge, on the site of the Romanian General Direction of Social Assistance and Child Protection [31].

### 2.3. Statistical Analysis

We employed the G*Power 3.1.9.2 software to calculate the corresponding achieved power for each key outcome criterion of our study (MMSE, ADL, and GDS-15), to establish if our sample size was large enough to obtain statistically significant results, and we obtained values of over 90% for each of them. For MMSE, we obtained an effect size of −1.80 for the corresponding sample sizes of 103 and 87 subjects, and thus the achieved power was 100%. For ADL, and an effect size = −0.52, the achieved power was 94.44%, and for GDS-15 and an effect size = 0.80, the G*Power 3.1.9.2 software calculated an achieved power of 99.98%. The results are presented as percentages for categorical variables and as mean values ± standard deviation for continuous data. The evaluation of blood test results, HR, SBP, DBP, IMT, ABI, and LV function parameters, and the interpretation of the results of the neuropsychological scales, were performed by using the unpaired *t*-test considering the two groups of patients. To analyze gender and age differences between groups A and B, we employed the impaired *t*-test. Similarly, the Chi-squared test was applied to determine differences between the categorical variables. Correlations between key data were performed by using the Spearman correlation coefficient. Bonnett and Wright’s (2000) method was used to estimate the corresponding 95% confidence intervals (95% CI) for the correlation coefficients [32]. To determine the impact of age, BP, HR, LVEF, BMI, eGFR, ADL, and GDS-15 on MMSE values, we build four logistic regression models, considering the forward method which has from the Wald test, to identify the most significant variables for which the highest odds ratio (OR) with a corresponding 95% CI were obtained. The final models were assessed for sensitivity, specificity, positive predicted value (PPV), negative predicted value (NPV), and associated ROC curves with their corresponding area under the ROC curve. Statistical analysis was conducted by using IBM SPSS Statistics version 20.0 software for Windows, with a significance level of 0.05.

### 2.4. Ethics

The protocol of this study was approved by the Institutional Review Board of Municipal Emergency Hospital Timisoara, Romania, nr. E1518/17.03.2021, and conducted in conformity with the indications of the Declaration of Helsinki. All subjects signed at admission in the hospital the standardized informed consent form, as required by the Romanian Health Authority.

## 3. Results

Our study population encompassed 190 patients: 104 (54.70%) were women, and 86 (45.30%) were men, with a mean age of 71.34 ± 9.82 years. In total, 55 subjects (28.90%) were under 65 years old, and 135 (71.10%) were older than 65 years. According to the presence or absence of CHF, they were divided into groups A and B. There were no statistically significant differences concerning age, BMI values, and risk factors such as smoking and obesity between groups A and B; see Table 1.

The most frequently encountered CV pathology in all 190 patients was SH, diagnosed in 154 (81.10%) of them; DM-2 was diagnosed in 57 (30.00%) subjects, hyperlipidemia in 101 (53.20%); 56 (29.50%) subjects were smokers and 29 (15.30%) were obese (Table 1).

In group A, there were included 62 (60.20%) women and 41 (39.80%) men, with a mean age of 71.99 ± 9.86 years, all of them diagnosed with CHF. None of these patients had NYHA functional class I; NYHA class II prevailed, being encountered in 85 (82.50%) subjects, while 15 (14.60%) had NYHA class III and 3 (2.90%) NYHA class IV. Concerning the underlying CV pathology, 99 of our patients (96.10%) were hypertensive, of which 14 (13.60%) had SH grade I, 43 (41.70%) SH grade II, and 42 (40.80%) had SH grade III; 53 (51.50%) of the subjects had CCS, and 58 (56.30%) participants AF, of whom 20 (19.40%) had paroxysmal AF, 11 (10.70%) persistent AF, and 27 (26.20%) permanent AF, and PAD was diagnosed in 10 (9.70%). Regarding co-morbidities, DM-2 was diagnosed in 37 (35.90%) subjects, 33 (32%) had chronic kidney (CKD) disease, 69 (67.00%) had hyperlipemia, 32 (31.10%) were smokers, and 20 (19.4%) were obese.

In group B, there were 42 (48.30%) women, and 45 (51.70%) men, with a mean age of 70.57 ± 9.77 years. SH was diagnosed in 55 (63.20%) patients, of which 27 (31.00%) had SH grade I and 28 (32.20%) had SH grade II. DM-2 was detected in 20 (23.00%) subjects, CKD in 13 (14.9%), hyperlipemia in 32 (36.80%), 24 (27.60%) were smokers, and 9 (10.3) were obese (see Table 1). We documented statistically significant differences between groups A and B, regarding the prevalence of SH (*p*-value < 0001), CKD (*p*-value = 0.006), and hyperlipemia (*p*-value < 0001).

Regarding laboratory data, there were no statistically significant differences between groups A and B (see Table 2), except for a slight decrease in Hemoglobin (Hb) and thrombocyte levels. There were no statistically significant differences between groups A and B, regarding low-density lipoprotein (LDL) cholesterol, high-density lipoprotein (HDL) cholesterol, and triglyceride (TG) levels.

Regarding CV risk factors, we documented statistically significant higher levels of creatinine (*p*-value = 0.003) and creatine kinase MB (CKMB) (*p*-value = 0.038), and lower sodium (*p*-value = 0.023) in patients from group A, compared to those from group B (see Table 2). It should be mentioned that although the values of eGFR were lower in group A than in group B, there were no statistically significant changes between the two groups (see Table 2). Regarding other biological parameters, no statistical discrepancies between the two patients’ groups were documented (see Table 2). The mean value of NT-pro-BNP levels in patients from group A was 2926.28 ± 807.194 pg/mL.

Concerning the severity of SH, the mean value of SBP (134.85 ± 21.77mm Hg) was statistically significantly higher in group A in comparison to group B (128.16 ± 16.60 mmHg), with a *p*-value = 0.017. The mean value of DBP (80.34 ± 14.49 mmHg) was also statistically significantly higher in patients from group A compared to those from group B, at 75.63 ± 11.09 mmHg (*p*-value = 0.021). The mean value of HR was statistically significantly higher in group A (76.61 ± 16.70 b/min) compared to group B (71.85 ± 11.55 b/min) (*p*-value = 0.022) (see Table 3).

The values of IMT measured on the left and on the right CCA were statistically significantly higher in patients from group A, compared to those assessed in patients from group B, Table 3. Although the values of ABI measured in subjects from group A were slightly lower than in group B, they were still in the normal range and we did not document statistically significant differences between the two groups.

These changes in blood pressure and IMT mean that alterations due to subclinical atherosclerosis were more evident in group A than in group B. The elevation of SBP and DBP in patients from group A may be attributed to a longer history of SH than in patients from group B.

Concerning the results of 2D-mode TTE evaluation, we detected statistically significantly higher values of the interventricular septum (IVS) thickness (12.42 ± 2.13 mm) in group A compared to group B (11.60 ± 1.98 mm) (*p*-value = 0.007), but without statistical significance regarding LV posterior wall (LVPW) thickness. Although we documented enlarged left atrium (LA) measurements in both groups, there was no statistically significant difference between them. LV end-systolic volume (LVESV) and systolic pulmonary arterial pressure (SPAP) were statistically significantly higher in group A compared to group B (*p*-value = 0.047 *p* = 0.041) (see Table 3).

LVEF was statistically significantly lower (55.91 ± 7.37%) in patients from group A compared to those from group B (59.14 ± 6%) (*p*-value = 0.001). We documented an LVEF < 50% in 16 (15.5%) patients from group A.

Regarding the results of the neuropsychological tests, in group A, 45 (43.7%) patients had MMSE scores above 27 points, 19 (18.4%) had MMSE scores between 24 and 27 points, signifying the presence of CD, and 26 (25.2%) had MMSE scores under 24 points, indicating dementia.

In group B, the number of subjects with signs of mild CD or dementia was lower, so there were 11 (12.6%) patients with MMSE scores between 24 and 27 points, 10 (11.5%) with MMSE under 24 points, and the remaining 66 (75.9%) patients had MMSE scores over 27 points, thus indicating the absence of CD.

We noticed that the parameters of all five neuropsychological scales were more severely altered in patients from group A compared to those from group B: MMSE and MoCA values statistically significantly decreased (*p*-value = 0.003 and *p*-value = 0.017, respectively), indicating a more severe CD, as well as for the parameters assessing the daily activity of patients, like ADL and IADL (*p*-value < 0.001 and *p* = 0.021, respectively), while parameters indicating depression, like GDS-15, had statistically significantly higher values in group A compared to group B (*p*-value = 0.015); see Table 4.

We noticed several sex-related differences in our study group. In group A, LVEDV was statistically significantly higher in men compared to women (*p* = 0.002). LVESV and sPAP were statistically significantly lower in male patients compared to female patients (*p*-value = 0.034 and *p*-value = 0.001, respectively). IMT values at the level of both carotid arteries were statistically significantly higher in female patients from group A than in those from group B (left IMT (mm) *p*-value = 0.006; right IMT (mm) *p*-value = 0.007). IVS, LVEDD, LVESV, and sPAP were statistically significantly higher (*p*-value = 0.008; *p*-value = 0.003; *p*-value < 0.001; and *p*-value = 0.005, respectively) while LVEF and total cholesterol were statistically significantly lower in women from group A compared to those from group B (*p*-value = 0.002 and *p*-value = 0.013, respectively). Regarding female patients, MMSE was statistically significantly lower (25.61 ± 5.11) in group A compared to group B (27.86 ± 3.21, *p*-value = 0.007). MoCA and ADL were also statistically significantly lower (*p* = 0.016 and *p* = 0.033, respectively), and GDS-15 was statistically significantly higher in women from group A than from group B (*p*-value = 0.002). Regarding male patients, ADL, IADL, and eGFR were statistically significantly lower (*p*-value = 0.005; *p*-value = 0.045; and *p*-value = 0.018, respectively); SBP, HR, and creatinine were statistically significantly higher in group A compared to group B (*p*-value = 0.034; *p*-value = 0.039; and *p*-value = 0.009, respectively).

Regardless of gender, this means that patients with a very high CVRP, and associated CHF, may have more severe cognitive impairment, slightly altered daily activity, associated depression, as well as more significant signs of subclinical atherosclerosis, compared to those without CHF.

Considering patients’ age, in group A, there were 27 (26.20%) patients aged under 65 years with a mean age of 59.93 ± 5.98 and 76, 73.80%, with a mean age of 76.28 ± 6.99, years. In group A, MMSE was statistically significantly lower (24.75 ± 5.40) in patients over 65 years compared to the younger subcategory, who had a value of 27.63 ± 2.72 (*p* = 0.009). MoCA was statistically significantly lower, at 22.79 ± 6.43, in patients in group A and over 65 years, compared to those under 65 years, who had a value of 25.11 ± 4.01 (*p*-value = 0.033). GDS was statistically significantly higher 7.28 ± 2.21 in subjects over 65 years, compared to 6.11 ± 2.59 in those younger than 65 years (*p*-value = 0.025).

In group A, IMT measured at the right CCA was statistically significantly higher, 0.77 ± 0.24, in patients older than 65 years compared to 0.60 ± 0.29 in those under 65 years (*p*-value = 0.005). SBP (mmHg) was statistically significantly higher, 137.57 ± 21.36, in patients over 65 years compared to 127.22 ± 21.49 in patients younger than 65 years (*p*-value = 0.037). The other parameters evaluated using neuropsychological, hemodynamic, and ultrasonographic assessment showed more pronounced alterations in patients from group A, regarding age.

Our results show that patients with CHF aged over 65 years have an increased probability of developing CD and depression, as well as more advanced signs of subclinical atherosclerosis and the occurrence of CV or cerebrovascular events.

In group A, MMSE was statistically significantly lower in patients with CHF NYHA class III and IV (23.11 ± 6.39), compared to those with class II (26.01 ± 4.53), *p*-value < 0.05. We noticed statistically significant lower levels of MoCA in patients with NYHA class III and IV (20.28 ± 6.09), compared to individuals with class II (24.06 ± 5.75), *p*-value < 0.05.

Although our results showed that daily activity, expressed through ADL and IADL values, was reduced and that depression, assessed by GDS-15 levels, was more severe in patients with NYHA class III and IV compared to subjects with NYHA class II, the difference was not statistically significant.

In patients with CHF, considering LVEF, we noticed a mild positive statistically significant correlation with MMSE (*p*-value = 0.002) and a moderate one with ADL and IADL (*p*-value ˂ 0.001). LVFE had a moderate statistically significant but negative correlation with GDS-15 (*p*-value ˂ 0.001); see Table 5.

We determined that the LVEDD had a mild negative statistically significant correlation with ADL and a moderate one with MMSE and IADL (*p*-value ˂ 0.001), but correlated positively with GDS-15 (*p*-value ˂ 0.001); see Table 5. Similarly, regarding the NT-pro-BNP levels, we evidenced negative moderate statistically significant correlations only with MoCA (r = −0.391, 95% CI −0.549; −0.207, *p*-value < 0.001). We documented mild positive, but statistically significant correlations between NT-pro-BNP levels and ADL and GDS-15 (*p*-value = 0.008 and *p*-value = 0.006, respectively) and negative ones with MMSE and IADL (*p*-value = 0.005 and *p*-value = 0.014, respectively), see Table 5. We also obtained mild, but statistically significant positive correlations between NYHA class III and MMSE (*p*-value = 0.005), and GDS-15 (*p*-value = 0.045), and negative ones between NYHA class III and ADL (*p*-value = 0.003), and IADL (*p*-value = 0.005); see Table 5. We observed a mild negative correlation between NYHA class III and MoCA values (*p*-value < 0.001). These results indicated a direct relationship between CD and CHF, expressed through decreased LVFE, NYHA class, and increased NT-pro-BNP levels.

To estimate the prognostic factors for the occurrence of CD, quantified by an MMSE score between 27 and 24 points, or dementia (MMSE < 24 points), in patients with CHF, and to assess the odds ratio, we build logistic regression modeling for these subjects to identify the most significant predicting factors: age, BMI, BP, HR, eGFR. We used similar models to identify the impact of ADL and GDS-15. We employed a forward method derived from the Wald test to determine the most significant variables. Our model was assessed for sensitivity, specificity, PPV, NPV, and the associated ROC curve.

Logistic regression analysis evidenced statistically significantly higher odds of CD for increased age and higher DBP values when considering CHF patients. In these subjects, for an additional increase of a year in age, the odds of CD were higher by a factor of 1.084 (OR = 1.084, 95% CI 1.026; 1.146); see Table 6. For an additional increase of 1 mm Hg in DBP, the odds of an MMSE score between 27 and 24 points were higher by a factor of 1.077 (OR = 1.077, 95% CI 1.042; 1.114). BMI, SBP, HR, and eGFR were not statistically significant in patients with CHF.

**Figure 2:**
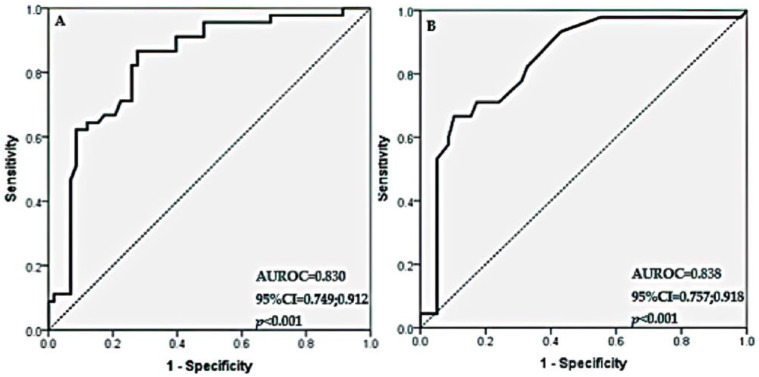
(**A**,**B**) Receiver Operating Characteristic (ROC) curve for multiple logistic regression model for association of MMSE under 27, but over 24, in patients with CHF with independent variables of (**A**) age, BMI, BP, HR, and eGFR; (**B**) ADL and GDS-15 scores.

We assessed the probability of MMSE between 27 and 24 points for patients with CHF by the following formula: exp(RS)/(1 + exp(RS)), where RS = −12.187 + 0.081 × (age) + 0.074 × (DBP). This model classified 73.79% of the patients correctly and had a sensitivity of 66.67%, a specificity of 79.31%, a PPV of 71.43%, and an NPV of 75.41%. The area under the ROC curve for this model was 0.830 (AUROC = 0.830, 95% CI 0.749; 0.912, *p*-value < 0.001); see Figure 2A.

To research the risk of having CD, characterized by an MMSE score between 27 and 24 points in patients with CHF, we built a logistic regression model to highlight the connection between LVEF and CD.

The odds of developing an MMSE score between 27 and 24 points were higher by a factor of 0.938 (OR = 0.938; 95% CI = 0.882; 0.998; *p* < 0.05), meaning that a decrease in LVEF of one percent was associated with a 6.2% increase in the odds of CD (MMSE between 27 and 24) in patients with CHF and preserved LVEF.

The odds of presenting CD were elevated by a factor of 0.928 (OR = 0.929; 95% CI = 0.885; 0.973; *p* < 0.05), meaning that a decline of 1% in LVEF was associated with 7.2% higher odds of developing an MMSE between 27 and 24 points in patients with CHF and preserved and reduced LVEF. Our results show that for each decrease in LVEF of one percent, the odds of being diagnosed with CD rise by 7.2% in subjects with CHF and reduced and preserved LVEF; see Table 7.

Our logistic regression analysis evidenced statistically significantly higher odds of CD for an increased GDS-15 score and statistically significantly lower odds of MMSE between 27 and 24 points for an increased ADL score when considering subjects from group A; see Table 8.

Logistic regression analysis highlighted statistically significantly higher odds of CD in patients with CHF. For an additional 1 unit in GDS-15 score, the odds of CD were higher by a factor of 1.349 (OR = 1.349, 95% CI 1.040; 1.750) (*p*-value < 0.05). In other words, an increase of 1 unit in GDS-15 is associated with an increase of 34.9% in the odds of having an MMSE < 27. We determined statistically significantly lower odds of an MMSE between 27 and 24 points for an additional unit of ADL score; the odds of CD were lower by a factor of 0.454 (OR = 0.454; 95% CI = 0.265; 0.777) (*p*-value < 0.05), so that an increase of 1 unit in the ADL score is associated with a 54.6% decrease in the odds of CD. This means that if new elements of depression occur (the GDS-15 score increased by 1 unit), the risk of cognitive impairment increases (with an increase of 34.9% in the odds of MMSE between 27 and 24 points), and if the quality of life is improved (the ADL score increased by 1 unit), the risk of CD decreases (with a 54.6% decline in the odds of an MMSE < 27).

The probability of CD for patients with CHF can be estimated by the following formula: exp(RS)/(1 + exp(RS)), where RS = 4.877 − 0.790 × (ADL) + 0.299 × (GDS). This model classified 79.61% of the patients correctly. The model had a sensitivity of 66.67%, a specificity of 89.66%, a PPV of 83.33%, and an NPV of 77.61%. The area under the ROC curve for this model was 0.838 (AUROC = 0.838, 95% CI 0.757; 0.918, *p*-value < 0.001); see Figure 2B.

Regarding an MMSE under 24 points, signifying dementia, the logistic regression analysis determined statistically significantly higher odds for MMSE to decrease to under 24 points for an increase in age, SBP, and HR; see Table 9. BMI, DBP, and eGFR were not statistically significant in patients with CHF.

The statistical regression analyses highlighted that in patients with CHF, the odds of dementia, characterized by a reduction in MMSE score to under 24 points, were more elevated for older patients, with higher SBP and HR values. For an additional elevation of one year in age, the odds of dementia were higher by a factor of 1.116 (OR = 1.116, 95% CI 1.040; 1.198) in CHF patients (*p*-value = 0.002). For any additional augmentation of 1mm Hg in SBP, the odds of dementia were higher by a factor of 1.046 (OR = 1.046, 95% CI 1.019; 1.074) in patients from group A (*p*-value = 0.001), and for an additional increase of 1 unit in HR, the odds of dementia were higher by a factor of 1.045 (OR = 1.045, 95% CI 1.011; 1.079) in CHF patients (*p*-value = 0.008). BMI, DBP, and eGFR were not statistically significant in patients with CHF.

**Figure 3:**
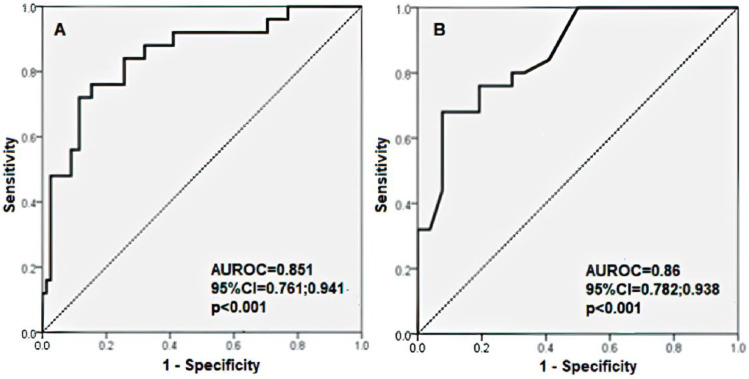
(**A**,**B**) Receiver Operating Characteristic (ROC) curve for multiple logistic regression model for association of MMSE under 24 in patients with CHF with independent variables (**A**) age, BMI, BP, HR, and eGFR; (**B**) ADL and GDS-15 scores.

The probability of developing an MMSE below 24 in patients with CHF can be estimated by the following formula: exp(RS)/(1 + exp(RS)), where RS = −19.166 + 0.110 × (age) + 0.045 × (SBP) + 0.044 × (HR). This model classified 82.52% of the patients correctly. The model had a sensitivity of 56.00%, a specificity of 91.03%, a PPV of 66.67%, and an NPV of 86.59%. The area under the ROC curve for this model was 0.851 (AUROC = 0.851, 95% CI 0.761; 0.941, *p*-value < 0.001); see Figure 3A.

Starting from the classification of CHF according to LVEF values, namely with reduced or preserved LVEF, we build a logistic regression model to identify the impact of LVEF (%) as a predicting factor for developing dementia (MMSE < 24 points) and to assess its odds ratio.

The logistic regression analysis highlighted statistically significantly higher odds of dementia for a decrease in the LVEF (%) when considering patients with preserved LVEF (*p*-value = 0.003); see Table 9. The odds of dementia were higher by a factor of 0.864 (OR = 0.864; 95% CI = 0.784; 0.952; *p* < 0.05), meaning that a decrease in LVEF of 1% was associated with 13.6% higher odds of developing an MMSE < 24 points in patients with CHF and preserved LVEF. This means that if the LVEF declines by 1%, the risk of dementia increases (with 13.6% higher odds of MMSE < 24 points) in subjects with preserved LVEF.

Considering all of group A, the odds of dementia were elevated by a factor of 0.949 (OR = 0.949; 95% CI = 0.901; 0.998; *p*-values = 0.042). In other words, a decrease of 1% in LVEF was associated with a 5.1% increase in the odds of developing an MMSE < 24 points in patients with CHF, both with preserved and reduced LVEF; see Table 10.

Regarding dementia, the logistic regression analysis underlined statistically significantly higher odds of an MMSE < 24 points for an increase in depression (GDS-15 score) and statistically significantly lower odds of an MMSE < 24 points for an increase in the activity of daily living (ADL score) when considering CHF patients (*p*-value < 0.05); see Table 11.

The logistic regression analysis highlighted statistically significantly higher odds of an MMSE < 24 points in CHF patients. For an additional 1 unit in GDS-15 score, the odds of dementia were higher by a factor of 1.629 (OR = 1.629, 95% CI 1.118; 2.373) (*p*-value < 0.05). In other words, an increase of 1 unit in the GDS-15 is associated with an increase of 62.9% in the odds of an MMSE < 24.

We observed statistically significantly lower odds of developing an MMSE < 24 points for an additional unit of ADL score. The odds of dementia were lower by a factor of 0.518 (OR = 0.518; 95% CI = 0.324; 0.828; *p*-value < 0.05); specifically, an increase of one unit of the ADL score is associated with a 48.2% decrease in the odds of an MMSE < 24 points. This means that if new elements of depression occur (the GDS-15 score increases by 1 unit), the risk of dementia (MMSE < 24 points) increases (with an increase of 62.9% in the odds of MMSE < 24 points), and if the quality of life is improved (the ADL score increases by 1 unit), the risk of dementia (MMS < 24 points) decreases (with a 48.2% decrease in the odds of an MMSE < 24 points).

The probability of dementia for patients with CHF can be estimated by the following formula: exp(RS)/(1 + exp(RS)), where RS = 0.845 − 0.658 × (ADL) + 0.488 × (GDS-15). This model classified 84.47% of the patients correctly. The model had a sensitivity of 60.00%, a specificity of 92.31%, a PPV of 71.43%, and an NPV of 87.80%. The area under the ROC curve for this model was 0.860 (AUROC = 0.860, 95% CI 0.782; 0.938, *p*-value < 0.001); see Figure 3B.

## 4. Discussion

In our study, we aimed to highlight the impact of various factors on the progression of CD in patients with CHF. Along with the recent increase in life expectancy of the global population, it has been estimated that around 65 million people are living with a diagnosis of CHF worldwide. While CHF was considered primarily a disease of older age, in recent decades, a rise in the number of younger individuals suffering from CHF has been noticed, probably due to an elevated prevalence of CV diseases, DM-2, obesity, and other metabolic and endocrine dysfunctions [33]. In our study, through an integrated approach between a cardiologist and a neurologist, we attempted to identify earlier the onset of cognitive impairment in patients with CHF. For this purpose, we employed five neurophysiological scales and highlighted a higher incidence and severity of CD in this category of patients. Another aim of our study was to evaluate the impact of depression and decreased self-care capacity, drawing attention to the bidirectional interdependence of these pathologies, with potential therapeutic and prognostic consequences. Concomitantly, several studies described a more accelerated decline of cognitive function in CHF patients, raising additional management and therapeutic issues [1,4]. CHF represents one of the main reasons for hospitalization and mortality [33], and since it is expected that the prevalence of CHF, CD, and dementia will increase in the following years, these pathologies are rapidly evolving into global healthcare concerns [34].

Considering the continuous augmentation of CD’s prevalence in patients with CHF, some studies have reported high percentages between 40 and 60% or even 80% [34,35]. Several meta-analyses were conducted on this topic. For example, the results of four longitudinal studies indicated that the risk ratio of developing dementia associated with CHF was 1.80 (95% CI, 1.41–2.31) [36]. As the incidence of CD is age-related, it being encountered more frequently in older patients [37], several studies did not consider the analysis of CD in subjects younger than 45 years [25,38,39]. As evidenced very well in their research based on the Rotterdam study, van der Wilik et al. firstly excluded subjects under 55 years, but due to a decrease in the age of patients diagnosed with CD, in their subsequent selection from 2006, they decreased the age barrier to 45 years [40]. In our study, by analyzing the factors influencing the occurrence of CD, and evaluating its severity by employing five neuropsychological scales in patients with and without CHF, we noticed that individuals over 65 years old and of the female gender prevailed, and although there were no statistically significant differences concerning age, BMI, smoking history, and biological parameters, excepting the lipid profile, between patients with and without CHF, the first group generally had more comorbidities and a more severe alteration in cognitive function, with this having been assessed in more than half of patients. Concerning the lipid profile, we noticed statistically significant lower values of total cholesterol in patients with CHF compared to those without (*p*-value = 0.015), probably due to a more intense cholesterol-lowering treatment in the first category. These patients also had decreased values of Hb and thrombocytes, possibly caused by the concomitant anticoagulant therapy in subjects with AF, a pathology encountered only in this group, but also due to the increased prevalence of CKD. By employing the multi-logistic regression analysis, our data indicated that age and BP values were significant influential factors for the decline in cognitive function in our study group.

The results of Sterling et al. suggest that CD usually develops after the occurrence of CHF and not before [5,40,41], although the responsible pathophysiological mechanisms are largely unknown. It is assumed that underlying pathologies, especially SH, DM-2, CCS, and AF, play an essential role. In a study published by Moneo et al., CD was diagnosed in 145 patients (27.6%) out of 525 subjects with CHF. Hypertensive patients and those with an ischemic etiology of CHF (OR:1.99, 1.25–3.17, *p*-value = 0.004) presented a greater impairment in cognitive function, compared to those with other causes of CHF [42]. Patients with CHF and CD were older, and the female gender prevailed, especially in patients with an ischemic etiology of CHF [OR: 2.303, (95% CI 1.204–4.405, *p*-value < 0.001). In our study, we also observed more frequently the concomitance of SH, CCS, AF, and DM-2. When evaluating patients’ cognitive function, we observed statistically significantly more altered levels of MMSE and MoCA, with more reduced daily activity and more severe depression in patients with CHF. Similar observations have also been presented in other research [43].

The development of CD in patients with CHF is a continuous and progressive process. In his study, Adelborg et al. followed up his patients over 35 years, and observed that subjects with CHF have a 1.5-times-higher risk of developing vascular dementia, and a 1.3-times-higher risk of developing other forms of dementia [5,44]. In another study, published by Hammond et al., a decrease in MMSE of 10.2 points [95% CI, 8.6–11.8] was observed in 496 patients with CHF during a 5-year follow-up period, compared to a decrease of almost half in MMSE scores of approximately 5.8 points [95% CI, 5.3–6.2] in control patients during the same interval of time, but without a statistical difference between the subjects with reduced or preserved LVEF [5,45].

Several studies have noticed that CD is a common pathology encountered in subjects with CHF and have suggested that this association affects the quality of life and significantly increases the hospitalization rate and mortality of these patients [3,35].

In a study published by Holm et al. that evaluated 281 patients with CHF, the MoCA scale was used to assess CD. Patients with an MoCA score < 23 points had a prevalence of 29%; they were associated with a higher risk of mortality (OR = 2.17; CI; 1.34–3.50; *p*-value = 0.002) and of readmission to hospital (OR = 1.62; CI; 1.14–2.29; *p*-value = 0.007). Instead, an MoCA score < 26 points was associated with a risk of rehospitalization, but not with a rise in mortality. Thus, in this study, it was observed that patients with CHF and a lower MoCA score have a higher risk of rehospitalization, and those with CHF and global cognitive impairment and attention deficit had a higher risk of mortality after discharge from hospital independently of the presence of CD [46]. Thus, in patients with CHF, the presence of CD can be considered a risk factor for mortality and rehospitalization [7].

In the last year, attention has been given to the significance of NT-pro-BNP in evaluating the risk of developing CD in patients with and without CV pathology. Several review articles have indicated that elevated NT-pro-BNP levels could represent a risk factor for altered cognition [3,5]. The Rotterdam study followed up 9566 individuals for a median period of 5.5 years, and determined a direct correlation between elevated NT-pro-BNP levels and the severity and evolution of CD, but there was no relationship with the damage of the brain structure, as assessed by MRI [47]. Similarly, in our research, we highlighted statistically significant negative correlations between NT-proBNP levels and cognitive function assessed by MMSE, but also with the severity of depression and the impairment of daily activity. The Irish Longitudinal Study on Ageing (TILDA) followed 4105 individuals for 10 years and concluded that NT-pro-BNP levels could be used as a predictor for CD in the elderly, independently of associated CV diseases [17]. Other studies that analyzed the connectivity in the cuneus revealed an association between NT-pro-BNP values and a decrease in the centrality of the brain [48,49].

Another important aspect observed in patients with CHF and CD was the increased tendency to develop associated depressive symptoms, the prevalence of depression being higher among patients with CD (*p*-value = 0.064) [41], rendering them more frail (OR:1.58, CI; 0.99 to 2.50, *p*-value = 0.050), and increasing their chances to require a caregiver to help them to take care of themselves and to medicate them [42].

In a review published by Maura Leto et al., it was stated that in elderly patients with CHF, depression can be considered as an additional risk factor. In patients with CHF, depression plays an interactive role in determining changes in cognitive function. Multiple pathophysiological mechanisms, observed both in the depressive illness and in cognitive impairment, such as hypoperfusion at the level of cerebral white matter or a reduction in cerebral gray matter in cortical and subcortical regions, associated with endothelial dysfunction and precocious atherosclerosis in the cerebral vasculature, are responsible for the development of neuroanatomical structural changes [50]. All these processes are favored by CHF and other underlying CV diseases or associated pathologies. Another link between depression, cognitive disorders, and CHF seems to be related to alterations of neurohormone levels such as cortisol or to the presence of pro-inflammatory cytokines (IL-6, TNF-α, C-reactive protein) [51].

In a study published by Oud et al. regarding the evaluation of CD and depression in 157 patients with CHF, it was evident that cognitive impairment, defined by an MoCA score < 22 points, was present in 56 (36%) of the patients; elements of depression characterized by a GDS-15 score > 5 points were present in 21 (13%) subjects with CHF, and of these, 6 also had associated cognitive disorders. From the total number of patients in the study, 109 (69%) had an MoCA score < 26 points (18). It is worth mentioning that in this study [52], during a 6-month follow-up, 44 (28%) patients were readmitted to the hospital, of which 24 (15%) were due to CHF. In the readmitted patients, there were no differences related to the presence or absence of cognitive disorders (*p*-value = 0.62 and *p*-value = 0.84, respectively) or depression (*p*-value = 0.95 and *p*-value = 0.61, respectively). Cognitive impairment and depressive symptoms were not associated with mortality (*p*-value = 0.83 and *p*-value = 0.24, respectively) [52].

As documented in our study, it appears that there is a correlation between CD and depression in patients with CHF. By employing multivariate regression analysis, we demonstrated a strong association between cognitive impairment, both in patients with CD (MMSE between 24 and 27 points), and also in those with dementia (MMSE under 24 points) and depression, assessed by the GDS-15 scale, and their ability to care for themselves, as discerned by the ADL scale. Logistic regression analysis revealed statistically significantly higher odds of CD in patients with CHF. Therefore, in patients with CHF and for whose MMSE score fell in a certain range—24 ˂ MMSE ˂ 27 points—, considering the results of the GDS-15 score, the odds ratio was 1.349 (OR = 1.349, 95% CI 1.040; 1.750, *p*-value < 0.05), while in those with an MMSE < 24 points, the odds were even higher (OR = 1.629, 95% CI 1.118; 2.373, *p*-value < 0.05). We obtained similar results concerning the ADL score (OR = 0.454; 95% CI = 0.265; 0.777, *p*-value < 0.05, and OR = 0.518; 95% CI = 0.324; 0.828; *p*-value < 0.05, respectively).

Patients with CHF and CD tend to have a lower level of self-care, manage everyday activities with difficulty, have a lower level of self-confidence, and show a decrease in adherence to medication, which may favor CHF decompensations, thus leading to frequent hospitalizations and increasing mortality and morbidity. A meta-analysis published by Lovell et al. [53] regarding the evaluation of factors influencing the self-care capacity of patients with CHF and CD highlighted the fact that subjects with CHF and an MMSE < 24 points and MoCA < 24 points had lower self-care scores than patients with an MMSE > 24 points or MoCA > 24 points, respectively. Male patients and those with associated comorbidities such as DM-2 or depression had lower IADL scores. Also, patients with severe CHF presented a lower degree of self-care. Patients with CHF, SH, AF, and a history of major CV events had lower MMSE scores than those without [53].

Therefore, considering the negative impact of CD in patients with CHF, the early diagnosis and prompt management of cognitive impairment are of the utmost importance. The efficient screening of patients with CHF for the onset of precocious signs suggestive of CD, by employing the MMSE and MoCA scales, two easy-to-use global cognitive screening tools, is essential in detecting patients with memory disorders [5].

There is little information regarding the prevention and management of CD in patients with CHF. Optimizing their treatment and correcting risk factors are important methods of primary approaches for the prevention of CD. Physical activity is an important non-pharmacological method of preventing CD, with a significant effect on cerebral neuroprotection in patients with cognitive impairment. It was observed that physical activity influences postural balance [54].

The presence of CD in patients with CHF can affect their daily activity and implicitly the ability to move and exercise. In these individuals, lifestyle changes are particularly important, including a progressive increase in exercise and dietary adaptations, which can improve memory. The increase in the number of daily steps predicts better cognitive function, with beneficial effects on the thalamus and the increase in the subcortical volume. Physical exercises and diet modification, such as salt reduction, and a high-fiber or Mediterranean diet, may have beneficial effects in maintaining cognitive function and improving language, attention, and orientation abilities, thus reducing the risk of dementia [3]. It is particularly important to screen patients with CHF for CD, to manage the patients at risk of developing CD, and to identify those with mild forms of CD as quickly as possible to better manage and treat these patients to slow down or even stop the disease progression and improve its prognosis [55].

According to the European Society of Cardiology Guidelines regarding the comprehensive management of individuals with CHF and CD, a complex multidisciplinary approach by a CHF team is required, also including, in addition to cardiologists and neurologists, professionals providing personalized advice for self-care, medication management, and for the patient’s family support. All these aspects are particularly important for improving adherence to medication and for the complex care of patients with CHF and CD, gradually increasing their quality of life [39,45,56].

Study limitations: Our study has several limitations, the main one being that it is a cross-sectional, single-center study, conducted on a limited study population. Another limitation is the absence of a follow-up of our patients. A third limitation is that we did not analyze if there were differences regarding the severity of CD in CHF patients with reduced versus preserved LVEF, considering that our study group mostly included patients with CHF with preserved LVEF. A subsequent splitting of the study population would have limited the statistical significance of our results, but it could represent a topic of future research.

## 5. Conclusions

Patients with CHF manifest an increased probability of developing CD; its severity is also influenced by a more advanced age, the female gender, comorbidities, especially SH, and the magnitude of LVEF decline, even in patients with preserved levels. We documented a direct relationship between MMSE scores and LVEF and an indirect one between MMSE and NT-pro-BNP levels. The concomitance of depression and reduced activity levels are aggravating CD in these patients, suggesting a worse prognosis. Therefore, a more comprehensive evaluation of patients with CHF by a multidisciplinary team should be recommended to identify associated pathologies, especially cognitive and mental health issues, thus allowing the optimization of their management.

## Figures and Tables

**Table 1:** Patient demographic data, cardiovascular risk factors, and comorbidities.

Patients’ Characteristics	All Patients Included in the Study	Group A (103 P)—with HF	Group B (87 P)—without HF	*p*-Value Between Group A and B
Age (mean ± SD)	71.34 ± 9.82	71.99 ± 9.86	70.57 ± 9.77	0.323
Age ≤ 65 (mean ± SD)	59.58 ± 5.46	59.93 ± 5.98	59.25 ± 5.01	0.651
Age > 65 (mean ± SD)	76.24 ± 6.59	76.28 ± 6.99	75.95 ± 6.20	0.778
Age in men (mean ± SD)	69.78 ± 9.92	69.66 ± 10.51	69.89 ± 9.47	0.915 *
Age in women (mean ± SD)	72.63 ± 9.58	73.53 ± 9.17	71.31 ± 10.13	0.248 *
Men (%)	45.30	39.80	51.70	0.1
Women (%)	54.7	60.20	48.30	0.1
BMI (kg/m^2^)	26.79 ± 4.83	26.65 ± 4.52	26.97 ± 5.19	0.649
SH (%)	81.1	96.10	63.20	<0.001
SH grade I	21.6	13.6	31	0.004
SH grade II	37.4	41.7	32.2	0.175
SH grade III	22.1	40.8	-	-
AF (%)	30.5	56.3	-	-
Paroxysmal	10.5	19.4	-	-
Persistent	5.8	10.7	-	-
Permanent	14.2	26.2	-	-
DM-2(%)	30	35.90	23.00	0.053
CKD (%)	24.20	32.00	14.90	0.006
Smoking (%)	29.50	31.10	27.60	0.600
Hyperlipemia (%)	53.20	67.00	36.80	<0.001
Obesity (%)	15.30	19.40	10.30	0.083

Legend: AF: atrial fibrillation; BMI: body mass index; CKD: chronic kidney disease; DM-2: type 2 diabetes mellitus; P: patients; SD: standard deviation; SH: systolic hypertension. p-values calculated with unpaired *t*-tests (*) and with Chi-square tests.

**Table 2:** Biological parameters in patients from groups A and B (with/without CHF).

Biological Parameter	All Patients	Group A 103 Patients	Group B 87 Patients	*p*-Value
Hemoglobin (g/dL)	13.91 ± 1.99	13.88 ± 2.19	13.95 ± 1.73	0.809
Hematocrit (%)	41.55 ± 5.26	41.72 ± 5.73	41.36 ± 4.66	0.636
Thrombocytes × 10^3^/µL	225.08 ± 57.31	221.31 ± 58.79	229.56 ± 55.51	0.324
Total Cholesterol (mg/dL)	182.80 ± 58.76	173.34 ± 51.67	194.00 ± 64.71	0.015
LDL Cholesterol (mg/dL)	131.33 ± 50.85	129.08 ± 48.83	133.98 ± 53.30	0.510
HDL Cholesterol (mg/dL)	48.25 ± 16.98	46.93 ± 12.03	49.82 ± 21.38	0.264
TG (mg/dL)	131.10 ± 55.95	130.52 ± 49.50	131.79 ± 63.04	0.879
Glycemia (mg/dL)	109.09 ± 28.08	110.27 ± 35.04	107.70 ± 16.53	0.531
Creatinine (mg/dL)	0.99 ± 0.24	1.03 ± 0.24	0.93 ± 0.21	0.003
Urea (mg/dL)	40.64 ± 17.06	41.68 ± 17.69	39.40 ± 16.38	0.358
eGFR (mL/min.)	70.60 ± 19.12	69.15 ± 20.25	72.32 ± 17.66	0.257
ALT (U/L)	30.06 ± 19.10	28.06 ± 10.94	32.43 ± 25.49	0.140
AST (U/L)	26.15 ± 16.52	26.07 ± 11.06	26.24 ± 21.32	0.946
CKMB (U/L)	17.21 ± 9.35	18.50 ± 11.46	15.68 ± 5.66	0.038
Na (mmol/L)	140.82 ± 2.65	140.42 ± 2.69	141.30 ± 2.53	0.023
K (mmol/L)	4.10 ± 0.43	4.10 ± 0.42	4.11 ± 0.46	0.929
Uric acid (mg/dL)	6.25 ± 1.53	6.16 ± 1.35	6.36 ± 1.71	0.373
NT-pro-BNP (pg/mL)	-	2926.28 ± 807.194	-	-

Legend: ALT: alanine aminotransferase; AST: aspartate aminotransferase; CKMB: creatine kinase MB; eGFR: estimated glomerular filtration rate; HDL: high-density lipoprotein; K: potassium; LDL: low-density lipoprotein; Na: sodium; NT-pro-BNP: N-terminal pro–B-type natriuretic peptide; TG: triglycerides.

**Table 3:** Clinical and laboratory parameters in patients from groups A and B (with/without CHF).

Clinical and Laboratory Parameters	All Patients	Group A 103 Patients	Group B 87 Patients	*p*-Value
BMI (kg/m^2^)	26.79 ± 4.83	26.65 ± 4.52	26.97 ± 5.19	0.649
SBP (mmHg)	131.79 ± 19.81	134.85 ± 21.77	128.16 ± 16.60	0.017
DBP (mmHg)	78.18 ± 14.43	80.34 ± 14.49	75.63 ± 11.09	0.021
HR (b/min)	74.43 ± 14.73	76.61 ± 16.70	71.85 ± 11.55	0.022
Parameters assessing atherosclerosis				
IMT (mm) left	0.70 ± 0.25	0.73 ± 0.26	0.66 ± 0.25	0.048
IMT (mm) right	0.68 ± 0.29	0.72 ± 0.27	0.63 ± 0.30	0.031
ABI left	1.10 ± 0.16	1.09 ± 0.12	1.12 ± 0.20	0.159
ABI right	1.10 ± 0.13	1.09 ± 0.16	1.10 ± 0.10	0.708
Echocardiographic parameters				
IVS (mm)	12.05 ± 2.10	12.42 ± 2.13	11.60 ± 1.98	0.007
LA (mm)	41.42 ± 6.19	41.88 ± 6.01	40.88 ± 6.40	0.270
LVPW (mm)	12.23 ± 2.57	12.28 ± 2.61	12.17 ± 2.54	0.760
LVESD (mm)	24.50 ± 6.58	24.75 ± 7.21	24.19 ± 5.78	0.552
LVEDD (mm)	46.43 ± 5.28	47.09 ± 5.50	45.65 ± 4.92	0.061
LVESV (mL)	37.01 ± 14.71	38.96 ± 15.64	34.70 ± 13.27	0.047
LVEDV (mL)	76.98 ± 19.59	76.18 ± 18.62	77.93 ± 20.75	0.542
LVEF (%)	57.39 ± 6.95	55.91 ± 7.37	59.14 ± 6.00	0.001
sPAP (mmHg)	39.70 ± 11.49	41.26 ± 10.78	37.85 ± 12.09	0.041

Legend: ABI: ankle brachial index; BMI: body mass index; DBP: diastolic blood pressure; HR: heart rate; IMT—intima–media thickness; IVS: interventricular septum; LA: left atrium; LVEDD: left-ventricular end-diastolic diameter; LVEDV: left-ventricular end-diastolic volume; LVEF: left-ventricular ejection fraction; LVESD: left-ventricular end-systolic diameter; LVESV: left-ventricular end-systolic volume; LVPW: left-ventricular posterior wall; SBP: systolic blood pressure; sPAP: systolic pulmonary arterial pressure.

**Table 4:** Neuropsychological tests in patients from groups A and B (with/without CHF).

Neuropsychological Tests	All Patients	Group A 103 Patients	Group B 87 Patients	*p*-Value
MMSE (points)	26.34 ± 4.42	25.50 ± 5.00	27.3 ± 38.41	0.003
MoCA(points)	24.26 ± 5.42	23.40 ± 5.96	25.26 ± 4.53	0.017
ADL (points)	9.39 ± 1.06	9.15 ± 1.23	9.67 ± 0.74	0.000
IADL (points)	6.73 ± 1.76	6.47 ± 1.90	7.03 ± 1.54	0.021
GDS-15 (points)	6.61 ± 2.29	6.98 ± 2.36	6.18 ± 2.15	0.015

Legend: ADL: Activities of Daily Living Score; GDS-15: Geriatric Depression Scale 15 questions; IADL: Instrumental Activities of Daily Living Score; MMSE: Mini Mental State Examination Scale; MoCA: Montreal Cognitive Assessment Scale.

**Table 5:** Correlation between left-ventricular ejection fraction (LVEF), left-ventricular end-diastolic diameter (LVEDD), NT pro-BNP levels, and results of neuropsychological tests (MMSE, ADL, GDS-15, and IADL).

Parameter	MMSE	ADL	GDS-15	IADL
LVEF				
r	0.226	0.321	−0.321	0.311
95% CI	0.085; 0.358	0.184; 0.446	−0.438; −0.174	0.173; 0.437
*p*-value	0.002	˂0.001	˂0.001	˂0.001
LVEDD				
r	−0.324	−0.251	0.450	−0.383
95% CI	−0.449; −0.187	−0.382; −0.110	0.322; 0.562	−0.502; −0.250
*p*-value	<0.001	<0.001	<0.001	<0.001
NT-pro-BNP				
r	−0.275	−0.258	0.269	−0.242
95% CI	−0.448; −0.082	−0.433; −0.063	0.076; 0.442	−0.418; −0.048
*p*-value	0.005	0.008	0.006	0.014
NYHA class III				
r	−0.204	−0.217	0.145	−0.202
95% CI	−0.338; −0.062	−0.35; −0.075	0.002; 0.282	−0.336; −0.06
*p*-value	0.005	0.003	0.045	0.005

Legend: ADL: Activities of Daily Living Score; GDS-15: Geriatric Depression Scale 15 questions; IADL: Instrumental Activities of Daily Living Score; LVEDD: left-ventricular end-diastolic diameter; LVEF: left-ventricular ejection fraction; MMSE: Mini Mental State Examination Scale; NT-pro-BNP: N-terminal pro–B-type natriuretic peptide; NYHA: New York Heart Association; Spearman’s correlation coefficient.

**Table 6:** Multiple logistic regression analysis for risk of MMSE between 27 and 24.

Variable	Patients with 24 ˂ MMSE < 27 Points (%) *	OR (95% CI)	*p*-Value
Patients with CHF			
Age	45 (43.69%)	1.084 (1.026; 1.146)	0.004
DBP (mmHg)	45 (43.69%)	1.077 (1.042; 1.114)	<0.001

Legend: MMSE: Mini Mental State Examination Scale; OR = odds ratio, 95% CI = 95% confidence interval. * Percentages based on the total number of patients with CHF (103 patients).

**Table 7:** Multiple logistic regression analysis for risk of MMSE between 27 and 24 points.

Variable	Patients with MMSE Between 27 and 24 Points	OR (95% CI)	*p*-Value
Patients with CHF and preserved LVEF			
LVEF (%)	63 (36.20%)	0.938 (0.882; 0.998)	0.043
Patients with CHF (preserved and reduced) LVEF			
LVEF(%)	75 (39.47%)	0.928 (0.885; 0.973)	0.002

Legend: LVEF: left-ventricular ejection fraction; MMSE: Mini Mental State Examination Scale. OR = odds ratio; 95% CI = 95% confidence interval.

**Table 8:** Multiple logistic regression analysis for risk of MMSE < 27.

Variable	Patients with MMSE < 27 Points (%) *	OR (95% CI)	*p*-Value
Patients with HF			
ADL	45 (43.69%)	0.454 (0.265; 0.777)	0.004
GDS-15	45 (43.69%)	1.349 (1.040; 1.750)	0.024

Legend: ADL: Activities of Daily Living Score; GDS-15: Geriatric Depression Scale 15 questions. Note: OR = odds ratio, 95% CI = 95% confidence interval. * Percentages based on the total number of patients with CHF.

**Table 9:** Multiple logistic regression analysis for risk of MMSE < 24.

Variable	Patients with MMSE < 24 Points (Dementia) (%) *	OR (95% CI)	*p*-Value
Patients with CHF			
Age (years)	25 (24.27%)	1.116 (1.040; 1.198)	0.002
SBP (mmHg)	25 (24.27%)	1.046 (1.019; 1.074)	0.001
HR(b/min)	25 (24.27%)	1.045 (1.011; 1.079)	0.008

Note: HR: heart rate; MMSE: Mini Mental State Examination Scale; SBP: systolic blood pressure; OR = odds ratio; 95% CI = 95% confidence interval. * Percentages based on the total number of patients with CHF.

**Table 10:** Multiple logistic regression analysis for risk of MMSE < 24.

Variable	Patients with MMSE < 24 Points	OR (95% CI)	*p*-Value
Patients with CHF and preserved LVEF			
LVEF (%)	32 (18.4%)	0.864 (0.784; 0.952)	0.003
Patients with CHF (preserved and reduced) LVEF			
LVEF (%)	36 (18.9%)	0.949 (0.901; 0.998)	0.042

Legend: LVEF: left-ventricular ejection fraction; MMSE: Mini Mental State Examination Scale. OR = odds ratio; 95% CI = 95% confidence interval.

**Table 11:** Multiple logistic regression analysis for risk of MMSE < 24.

Variable	Patients with MMSE < 24 Points (Dementia) (%) *	OR (95% CI)	*p*-Value
CVRF Patients with HF			
ADL	25 (24.27%)	0.518 (0.324; 0.828)	0.006
GDS-15	25 (24.27%)	1.629 (1.118; 2.373)	0.011

Note: ADL: Activities of Daily Living Score; GDS-15: Geriatric Depression Scale 15 questions; MMSE: Mini Mental State Examination Scale; OR = odds ratio; 95% CI = 95% confidence interval. * Percentages based on total number of patients with CHF (103 patients).

## Data Availability

The data are available upon request to the first author, M.T.
